# Prevalence and predictors of falls in a health‐seeking older population: An outpatient‐based study

**DOI:** 10.1002/agm2.12096

**Published:** 2020-01-19

**Authors:** Manicka Saravanan Subramanian, Vishwajeet Singh, Prashun Chatterjee, Sada Nand Dwivedi, Aparajit Ballav Dey

**Affiliations:** ^1^ Department of Geriatric Medicine All India Institute of Medical Sciences New Delhi India; ^2^ Department of Biostatistics All India Institute of Medical Sciences New Delhi India

**Keywords:** fall risk, falls, falls screening, older people

## Abstract

**Background:**

Falls are one of the major causes of disability in older people. A wide range of risk factors for falls are described according to setting – inpatient, nursing homes and community. The aim of this study was to identify the risk factors for falls in an outpatient setting.

**Methods:**

In this cross‐sectional observational study, 160 consenting subjects were enrolled randomly, from the Geriatric Medicine outpatient department, All India Institute of Medical Sciences, New Delhi, India. Non‐ambulatory, seriously ill subjects were excluded. The subjects underwent brief evaluation including falls and geriatric assessment. They were grouped into fallers and non‐fallers. A multivariable logistic regression analysis was used to identify the factors associated with falls.

**Results:**

The prevalence of falls was 23.75% (38/160). Women were proportionately higher (26.31%) in the fallers group vis‐à‐vis 19.67% in the non‐fallers group. After multivariate analysis, opioids (odds ratio [OR] 5.24 [95% CI, 2.0 18‐13.611]), vision impairment (OR 2.71 [95% CI, 1.050‐07.011]), fear of falling (OR 3.17 [95% CI, 1.167‐08.629]), instrumental activity of daily living (IADL) impairment (OR 3.41 [95% CI, 1.251‐09.301]), anti‐anginal medications (OR 8.90 [95% CI, 0.997‐79.564]) and self‐employment (OR 5.37 [95% CI, 1.058‐27.329]) were associated with falls. Adequate nutrition (OR 0.82 [95% CI, 0.688‐00.976]) and caregiver support (OR 0.46 [95% CI, 0.275‐00.801]) were protective of falls.

**Conclusion:**

We identified the multi‐factorial etiology of falls. Patients having any of the above risk factors should undergo detailed fall risk assessment and preventive measures afterwards.

## 1 INTRODUCTION

Falls are defined as an event which results in a person coming to rest inadvertently on the ground or floor or other lower level.^1^ The Global Burden of Disease Study 2016 designated falls as an age‐related disease.^2^ Falls accounted for 678 000 deaths and are one of the top 30 causes of disability‐adjusted life years (DALYs) lost. The worldwide loss amounts to 21 million DALYs in men and 15 million DALYs in women, respectively. Notably, falls were one of the two leading causes of DALYs in women.^3^ The World Health Organization (WHO) states that adults >65 years were more commonly suffering from falls and >80% of these falls happen in low‐ and middle‐income countries. These countries also account for >80% fall‐related fatalities.^1^

The prevalence of falls varies according to age groups ranging from 28%‐35% and 32%‐42% in age groups of >65 and >75 years, respectively.^4^ It was also noted that there was significant variation in fallers according to age, gender, institutional care and community‐dwelling status.^4^ The existence of cross‐cultural differences leads to variations in many of these risk factor differences, which were not taken into account in many of the studies.^5^ Further, in India, the prevalence of falls reported varies between 18% and >50%. These differences from global reporting may be due to the cross‐country differences, heterogeneity of the population and the methodology used in the studies.^6^

Accordingly, the current study aimed to identify the prevalence of falls in the health‐seeking older population and the factors associated with falls.

## 2 MATERIALS AND METHODS

### 2.1 Study population and design

This was a cross‐sectional observational study carried out in the outpatient department of the All India Institute of Medical Sciences (AIIMS), New Delhi, India from August 2015 to July 2017. A total of 160 ambulatory subjects who gave written informed consent and aged >60 years were included in the study. Non‐ambulatory subjects and subjects with severe functional impairment, defined as ability to perform only one ADL,^7^ were excluded from this study.

A semi‐structured interview in Hindi was carried out to identify socioeconomic status, personal history, medical history and medication use. Medications were identified by looking at the health records or by the blister packs. Polypharmacy was defined as taking more than or equal to five medications in the same month.^8^ Orthostatic hypotension was defined as a drop in systolic BP >20 mm Hg or diastolic BP >10 mm Hg within three minutes of standing from a lying down position. It was measured by the Omron 7310™ apparatus which uses the oscillometric method.^9^

Functional impairment was assessed by Barthel activity‐dependent daily living (BADL) and Lawton instrumental activity‐dependent daily living (IADL). Impairment in any one domain was considered as dependent. Tinetti performance‐oriented mobility assessment (POMA) and timed up and go test (TUG) was used for mobility assessment. Vision was assessed by Snellen chart and E charts for illiterates and hearing impairment by WHO grades.^10^, ^11^, ^12^, ^13^

Frailty index was used to identify the frail population, Montreal Cognitive Assessment (MoCA) for identifying cognitive impairment, and the mini nutritional assessment‐short form (MNA‐SF) was used to identify the nutritional status. For the assessment of depression, the Geriatric Depression Scale (GDS‐15 version) was used.^14^, ^15^, ^16^, ^17^

### 2.2 Statistical methods

Statistical analysis was carried out using statistical software Stata/SE version 14.2 (StataCorp LP). Qualitative variables of the study were described as absolute/relative frequency with percentage and quantitative variables by mean (standard deviation)/median (quartile range). To find the association between qualitative independent variables, the chi‐square test/Fisher's exact test was used. To assess the association between two quantitative variables, the Pearson/Spearman correlation coefficient was used. To find the difference in quantitative variables between groups, the *t* test/Wilcoxon test was used according to the distribution of the data. To find the factors associated with falls, stepwise multivariable logistic regression analysis was used. Variables which were found to be significant under crude association up to a level of 25% and/or clinically relevant were considered for the stepwise procedure. Calibration of predicted probability of the developed model was assessed by Hosmer‐Lemeshow (HL) test and specification error by link test. Discrimination ability of the developed model was evaluated using the area under the curve. Results were presented in the form of odds ratio with corresponding 95% confidence interval (CI). *P* value <.05 was considered as statistically significant.

## 3 RESULTS

Out of the 160 subjects, 38 (23.75%) experienced falls. The mean age was 74.47 (8.94) and about three‐quarters (73.68%) of them were males. For the purpose of analysis, the upper middle class was included in the middle class and the lower middle class was included in the lower class. Out of the 38 fallers, 9 (23.68%) belonged to the upper class, 20 (52.64%) belonged to the middle class, and 9 (23.68%) were lower class. Two‐thirds of the fallers, 25 (65.79%), were part of a joint family, 17 (44.74%) and 16 (42.11%) were dependent on their family members for their financial needs and pensioners, respectively. Approximately, <10% were smokers. Mean BMI of the fallers was 22.46 (4.52). Fear of falling was seen in 78.95% of fallers and 54.92% of non‐fallers. The details of the study variables according to fallers and non‐fallers are shown in Table 1.

**Table 1:** Demographic variables and health status of the study population

Variables	Non fallers (N = 122)	Fallers (N = 38)	*T*/*χ* ^2^ values	*P* value
Age				
Mean (SD)	74.75 (7.07)	74.47 (8.94)	0.19	.84
Sex				
Males	98 (80.33%)	28 (73.68%)	0.76	.38
Females	24 (19.67%)	10 (26.31%)		
Education				
Illiterate	18 (14.75%)	7 (18.42%)	0.30	.59
School education and above	104 (85.25%)	31 (81.58%)		
Socioeconomic class				
Upper class	37 (30.33%)	9 (23.68%)	3.61	.17
Middle class	71 (58.20%)	20 (52.64%)		
Lower class	14 (11.47%)	9 (23.68%)		
Caregiver status				
Living alone and widowers	22 (18.03%)	13 (34.21%)	4.44	.03
Joint family	100 (81.97%)	25 (65.79%)		
Financial status				
Dependent	55 (45.08%)	17 (44.74%)	3.24	.20
Independent – self‐ employed	6 (4.92%)	5 (13.16%)		
Independent – pensioners	61 (50%)	16 (42.11%)		
Alcohol use	25 (20.49%)	8 (21.05%)	0.63	.73
Smoking status				
Never a smoker	94 (77.05%)	27 (71.05%)	1.58	.43
Former smoker	24 (19.67%)	8 (21.05%)		
Smoker	4 (3.28%)	3 (7.89%)		
Body mass index				
Mean (SD)	23.51 (4.30)	22.46 (4.52)	1.29	.20
Fear of falling	67 (54.92%)	30 (78.95%)	7.01	.01
Diabetes	21 (17.21%)	10 (26.32%)	1.53	.21
Hypertension	66 (54.10%)	20 (52.63%)	0.02	.87
Joint pain	56 (45.90%)	19 (50%)	0.19	.66
Coronary artery disease	16 (13.11%)	8 (21.05%)	1.43	.23
Cerebrovascular diseases	7 (5.74%)	2 (5.26%)	0.01	>.99
Vision impairment (Cataract/glaucoma/refractory errors)	36 (29.51%)	17 (44.74%)	3.03	.08
Malignancies	3 (2.46%)	1 (2.63%)	0.00	>.99
Benign prostatic hypertrophy	28 (22.95%)	6 (15.79%)	0.89	.35
Hypothyroidism	7 (5.74%)	1 (2.63%)	0.59	.68
Respiratory diseases	13 (10.66%)	2 (5.26%)	0.99	.52
Urinary incontinence	6 (4.92%)	3 (7.89%)	0.48	.44
Total no of comorbidities				
Mean (SD)	2.36 (1.21)	2.74 (1.11)	−1.67	.09
Calcium channel blockers	43 (35.25%)	11 (28.95%)	0.51	.47
Angiotensin receptor blockers	38 (31.15%)	11 (28.95%)	0.07	.80
ACEi	3 (2.46%)	2 (5.26%)	0.75	.59
Diuretics	23 (18.85%)	2 (5.26%)	4.06	.04
Alpha blockers	27 (22.13%)	6 (15.79%)	0.71	.40
Anti‐histamines	8 (6.56%)	1 (2.63%)	0.84	.69
Beta‐blockers	24 (19.67%)	8 (21.05%)	0.03	.85
Thyroxine	8 (6.56%)	2 (5.26%)	0.08	>.99
Oral hypoglycemic agents	21 (17.21%)	10 (26.32%)	1.54	.21
Insulin	3 (2.46%)	0 (0%)	0.95	>.99
Antiplatelet agents	29 (23.77%)	13 (34.21%)	1.63	.20
Anti‐anginal medications	3 (2.46%)	3 (7.89%)	2.37	.15
Statins	29 (23.77%)	11 (28.95%)	0.41	.52
PPI/prokinetics/laxatives	67 (54.92%)	22 (57.89%)	0.10	.74
Nutritional supplements	69 (56.56%)	21 (55.26%)	0.02	.89
Opioids	33 (27.05%)	21 (55.26%)	10.31	<.01
Non‐opioid analgesics	41 (33.61%)	16 (42.11)	0.91	.34
Neuropathic medications	4 (3.28%)	5 (13.16%)	5.32	.03
Bronchodilators	17 (13.93%)	1 (2.63%)	3.70	.07
Antidepressants	15 (12.30%)	2 (5.26%)	1.51	.36
Anxiolytics	1 (0.82%)	1 (2.63%)	0.77	.42
Benzodiazepines	6 (4.92%)	0 (0%)	1.94	.34
No of medications				
Mean (SD)	4.92 (2.20)	5.13 (2.65)	−0.50	.62
Polypharmacy	68 (55.74%)	19 (50.00%)	0.38	.53
Orthostatic hypotension	17 (13.93%)	4 (10.53%)	0.29	.78
Frailty index				
Mean (SD)	0.22 (0.10)	0.27 (0.11)	−2.76	.01
Frail	53 (43.44%)	24 (63.16%)	4.80	.09
ADL				
Mean (SD)	19.55 (1.27)	19.34 (1.34)	0.86	.39
ADL impairment	32 (26.22%)	11 (28.94%)	1.54	.43
IADL				
Mean (SD)	0.28 (0.50)	0.31 (0.52)	−0.39	.69
IADL impairment	47 (38.52%)	21 (55.26%)	3.32	.09
Fall risk POMA				
Total score mean (SD)	19.77 (3.81)	18.89 (3.88)	1.32	.22
Low fall risk	17 (13.93%)	5 (14.16%)		
Medium fall risk	67 (54.92%)	15 (39.47%)	3.53	.17
High fall risk	38 (31.15%)	18 (47.37%)		
TUG score (s) Mean (SD)	13.69 (3.90)	14.66 (4.67)	−1.28	.20
MoCA				
Total score mean (SD)	21.63 (4.76)	20.50 (5.47)	1.23	.22
Mild cognitive impairment	51 (41.80%)	16 (42.11%)		
Dementia	25 (20.49%)	11 (28.95%)	1.54	.46
GDS				
Mean (SD)	3.65 (2.76)	4.13 (3.21)	−0.89	.37
Depressed	35 (35.68%)	14 (36.84%)	7.80	.09
Nutritional assessment				
MNA‐SF				
Total score Mean (SD)	10.74 (2.37)	9.47 (2.91)	2.72	.01
At risk of malnutrition	49 (40.16%)	11 (28.95%)		
Malnourished	60 (49.18%)	20 (52.63%)	2.43	.29

Abbreviations: ACEi, Angiotensin Converting Enzyme Inhibitors; ADL, Activity‐Dependent Daily Living; GDS, Geriatric Depression scale; IADL, Instrumental Activity‐Dependent Daily Living; MNA‐SF, Mini nutritional assessment short form; MoCA, Montreal cognitive assessment; POMA, performance‐oriented mobility assessment; PPI, Proton Pump Inhibitors; SD, Standard Deviation; TUG, Timed up and Go test.

Chronic conditions like joint pain (50%), diabetes (26.32%) and vision impairment (44.74%) were predominant in the fallers. Orthostatic hypotension was present in approximately 10% and there were not many differences in the number of comorbidities between the fallers and non‐fallers (2.36 [1.21] vs 2.74 [1.11]). Prokinetics and other gastrointestinal medicines (57.89%) were dominant in the fallers, followed by pain‐relief medicines like opioids and non‐opioid pain medicines (55.26%). The number of medications was higher in fallers 5.13 (2.65), and polypharmacy was less than the non‐fallers (55.74% vs 50%). Fallers were frailer (63.16%) and had poor functional status measured by impaired ADL (28.94%) and IADL (55.26%). On the evaluation of further fall risk by the Tinetti scale, fallers were at high risk of falls (47.37%) and poor mobility, higher fall risk in terms of TUG score 14.66 (4.67). Among fallers there was also more prevalence of dementia (28.95%), depression (36.84%) and malnourishment (52.63%) .

To identify the factors associated with falls, we took into account all the clinical, socioeconomic and demographic determinants. In univariate analysis, gender, dependent financial status, coronary artery disease, BMI, diabetes, oral hypoglycemic agents, fall risk, dementia, frailty, TUG score, total number of comorbidities, diuretics, antiplatelet agents, neuropathic medicines, and bronchodilators were also associated with falls. However, in the following multivariate analysis these factors were not significant.

Under multivariate analysis, opioids (OR 5.24 [95% CI, 2.018‐13.611]), vision impairment (OR 2.71 [95% CI, 1.050‐07.011]), fear of falling (OR 3.17 [95% CI, 1.167‐08.629]), IADL impairment (OR 3.41 [95% CI, 1.251‐09.301]), anti‐anginal medications (OR 8.90 [95% CI, 0.997‐79.564]), independent living‐ pension schemes (OR 1.06 [95% CI, 0.413‐02.736]) and self‐employment (OR 5.37 [95% CI, 1.058‐27.329]) were associated with higher risk of falls, while higher mini nutritional assessment score (OR 0.82 [95% CI, 0.688‐00.976]) and having a caregiver (OR 0.46 [95% CI 0.275‐00.801]) were found to be protective of falls (Table 2).

**Table 2:** Predictive model of falls from multivariable logistic regression analysis

Variable	*Z* values	OR (95% CI)
Opioids	3.40	5.24 (2.018‐13.611)
Anti‐anginal medications	1.96	8.90 (0.997‐79.564)
Fear of falling	2.26	3.17 (1.167‐08.629)
Vision impairment (cataract/glaucoma/refractive errors)	2.06	2.71 (1.050‐07.011)
IADL impairment	2.40	3.41 (1.251‐09.301)
MNA‐SF score	−2.24	0.82 (0.688‐00.976)
Independent living – pension schemes	0.13	1.06 (0.413‐02.736)
Independent living – self‐employed	2.03	5.37 (1.058‐27.329)
Caregiver status	−2.78	0.46 (0.275‐00.801)

Abbreviations: IADL, Instrumental Activity‐Dependent Daily Living; MNA‐SF, Mini Nutritional Assessment Short Form; OR, Odds Ratio.

**Figure 1:**
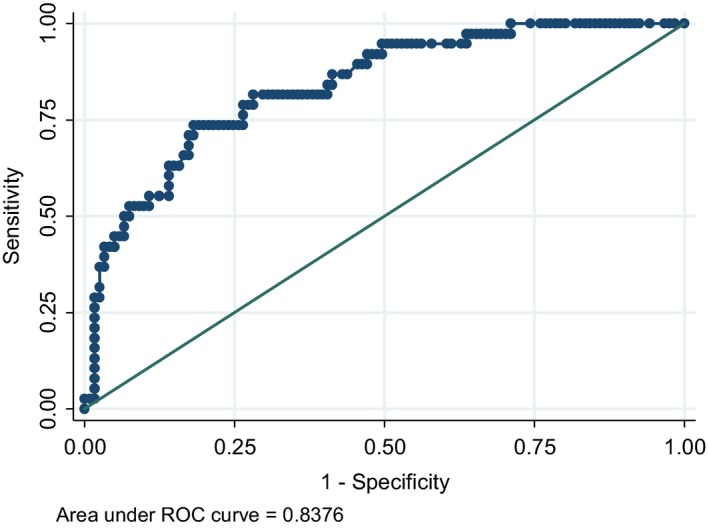
Area under the curve for the predictive model. Figure shows discrimination ability of the predictive model, ROC, Receptor operator curve

The discrimination ability of the developed model was found to be satisfactory and the model was able to discriminate a case of fall with probability 0.8376 (Figure 1).

## 4 DISCUSSION

In this observational study, the proportion of fallers was found to be 23.75%. The percentage of women in the fallers group (23.61%) was higher than in the non‐fallers group (19.67%), even though the prevalence of falls was a shade less than reported in the English longitudinal aging study (ELSA) (28.4% vs 23.75%). But ELSA also identified that women fall more than males.^18^ It was also reported that women tend to report falls more than male peers and seek health‐related advice in a medicare population.^19^ Subjects who cohabitate were less prone to falls in this study and falls were found to be unrelated to socioeconomic status, wealth, personal habits and body mass index. However, while the proportion of fallers was slightly higher in the poorest social strata and people who live alone, these findings may be due to the hospital setting, as these groups come to government hospitals. Similar findings were also observed in the ELSA study in the evaluation of incident falls.^18^ However, in the multivariate analysis, independent living was independently associated with falls and having a caregiver was found to be protective of falls. In the WHO global report on fall prevention,^20^ low socioeconomic status, poor standard of living and lack of caregiving were included in the fall risk model. It was proposed that the lack of access to health care resources in this population increases the risk of falls. This was partially due to the burden of chronic conditions and lack of health care and/or medication access.^21^

According to the traditional description, falls are related to number of co‐morbidities and number of medications. However, it was noted by Gale et al,^18^ that can be also confounded by various factors like anxiety, depression and immobility. In this group there was not much difference between the number of comorbidities in the two groups and polypharmacy was also widely prevalent. A study by Musich et al,^22^ found that in new and continuing fall‐related drug (FRD) users the fall rates were 7% and 8%, respectively, which also provides a platform for the polygenetic etiology of falls. In a study by Early et al,^23^ it was noted that fall rates increased with >6 FRDs in the 65‐74 age group and >5 FRDs in the 75‐84 age group. It should be duly noted that the mean age of the population in this study was around 74 years and the population was getting less than the number of medications, not limited to FRDs, required for a fall. This study is a hospital‐based cross‐sectional observational study and subjects may have received a prescription for an FRD only on the day of assessment and they may not have had the dose‐response relationship to experience the fall proposed by Gale et al.^18^

In the multivariate analysis, antianginals and opioids were significantly associated with falls. These findings were similar to the study by de Jong et al^24^ and may be due to sudden hypotension, arrhythmia or a syncopal episode.^25^ There was also a significant difference in fear of falls and vision impairment between the groups. In multivariate analysis also, they were found to be independently associated with falls. The complex interaction of postural control, visual gaze and anxious behavior were well explained by Young et al.^26^ Here the people with dementia had fallen more, even though no statistical significance was reached, and depression was also less prevalent in this population reflected by the mean scores.

In this study, frailty was not associated with falls. In a study by Li et al,^27^ it was found that for the frail population to experience one fall, the frailty index (FI) should be higher (22.15% experience one fall with FI 0.43). In this population, the FI was lower (0.22 non‐fallers vs 0.27 fallers). But the fallers had more instrumental activity‐dependent living (IADL) impairment (55.26% for fallers vs 38.52% in non‐fallers). In multivariate analysis IADL was also an independent predictor of falls. A study by Nourhashemi et al,^28^ found that IADL impairment was associated with a frailer population, fear of falling and associated with falls. Poor nutritional status was also contributing to falls and it was an independent predictor of falls. It was also shown by Chien et al.^29^that poor nutritional status was associated with falls and difficulties in IADL and it can also predict falls independently. 

The strengths of this study include an adequate sample size of the representative population, and extensive evaluation of a wide range of fall risk factors described in the literature. It also has certain limitations. It was a cross‐sectional study, so causality could not be established. There might be recall bias in recalling the incidence of fall. This was carried out in the outpatient department settings, as an interview.

## 5 CONCLUSION

Falls are one of the major public health problems. In this study, regardless of socioeconomic and demographic variations, the etiology of falls can be multifactorial. We need a large‐scale observational study to evaluate the fall risk factors, and design preventive as well as rehabilitative programs. However, the independent factors identified in this study can be used as a screening tool and older people who are having these factors may be subjected to detailed falls assessment and rehabilitation thereafter.

## CONFLICTS OF INTEREST

Nothing to disclose.

## AUTHOR CONTRIBUTIONS

*Writing of paper*: all authors. *Design, conceptualization literature review, review of medical records, data collection*: Subramanian. *Statistical analysis*: Singh. *Design, conceptualization and revision*: Chatterjee, Dwivedi and Dey.

## ETHICAL APPROVAL

All procedures performed in studies involving human participants were in accordance with the ethical standards of the institutional research committee (Institute ethics committee [IESC/T‐386/26.8.2015]) and with the 1964 Helsinki declaration and its later amendments or comparable ethical standards.
